# Young temperate tree species show different fine root acclimation capacity to growing season water availability

**DOI:** 10.1007/s11104-023-06377-w

**Published:** 2023-11-17

**Authors:** Florentin C. Jaeger, I. Tanya Handa, Alain Paquette, William C. Parker, Christian Messier

**Affiliations:** 1 Centre for Forest Research, Département des Sciences Biologiques, Université du Québec à Montréal, Montréal, QC Canada; 2 Forest Research and Monitoring Section, Ontario Ministry of Natural Resources and Forestry, Sault Ste. Marie, ON Canada; 3 Institut des Sciences de La Forêt tempérée, Université du Québec en Outaouais, Ripon, Canada

**Keywords:** Precipitation, Root necromass, Root functional classification, Root: shoot ratio, Root strategies

## Abstract

**Background and aims:**

Changes in water availability during the growing season are becoming more frequent due to climate change. Our study aimed to compare the fine-root acclimation capacity (plasticity) of six temperate tree species aged six years and exposed to high or low growing season soil water availability over five years.

**Methods:**

Root samples were collected from the five upper strata of mineral soil to a total soil depth of 30 cm in monoculture plots of *Acer saccharum* Marsh., *Betula papyrifera* Marsh., *Larix laricina* K. Koch, *Pinus strobus* L., *Picea glauca* (Moench) Voss and *Quercus rubra* L. established at the International Diversity Experiment Network with Trees (IDENT) field experiment in Sault Ste. Marie, Ontario, Canada. Four replicates of each monoculture were subjected to high or low water availability treatments.

**Results:**

Absorptive fine root density increased by 67% for *Larix laricina*, and 90% for *Picea glauca*, under the high-water availability treatment at 0–5 cm soil depth. The two late successional, slower growing tree species, *Acer saccharum* and *Picea glauca,* showed higher plasticity in absorptive fine root biomass in the upper 5 cm of soil (PIv = 0.36 & 0.54 respectively), and lower plasticity in fine root depth over the entire 30 cm soil profile compared to the early successional, faster growing tree species *Betula papyrifera* and *Larix laricina*.

**Conclusion:**

Temperate tree species show contrasting acclimation responses in absorptive fine root biomass and rooting depth to differences in water availability. Some of these responses vary with tree species successional status and seem to benefit both early and late successional tree species.

**Supplementary Information:**

The online version contains supplementary material available at 10.1007/s11104-023-06377-w.

## Introduction

Since the 1950s, climate change has led to increased mean annual precipitation globally as well as warmer temperatures and an increase in regional drought events caused by greater evapotranspiration (Masson-Delmotte et al. 2021). In many parts of the world, the changing climate has had a negative impact on forest productivity (Boisvenue & Running 2006). Even in regions that are not normally water limited, chronic drought in combination with other biotic and abiotic disturbance agents has emerged as an important global driver of recent increases in tree mortality (Hammond et al. 2022; Hartmann et al. 2022; Schuldt et al. 2020; Senf et al. 2020). Given the species-specific drought-mortality (Hammond et al. 2022; Hartmann et al. 2022; Schuldt et al. 2020; Senf et al. 2020), it is increasingly relevant to better understand acclimation potential of native tree species to changes in water availability to better predict drought tolerance and resilience of different tree species in the face of climate change (Allen et al. 2010; Choat et al. 2018).

Trees have evolved a variety of strategies to cope with drought (Brunner et al. 2015). For example, if the limiting resource is water, then plants allocate relatively more biomass into roots, and this can lead to increased root mass fraction and root to shoot ratios (Poorter et al. 2012). However, changes in root mass fraction in response to drought tend to occur only under prolonged and severe drought periods (Poorter et al. 2012). Furthermore, species adapted to dry conditions show stronger responses to drought in terms of biomass investment into longer lasting and deeper reaching roots than species from mesic climates (Brunner et al. 2015). Therefore, it is important to identify species which show plastic responses to changes in the environment and develop predictors which can identify the type of species most likely to display such responses (Nicotra et al. 2010).

Contrary to the above-mentioned whole tree analyses, relative measures of biomass allocation, and total fine root biomass in particular, tend to be lower under drier conditions as a result of reduced transpiration and respiration rates (Brunner et al. 2015). This has been clearly indicated in several recent meta-analyses (Brunner et al. 2015; P. Wang et al. 2020; Zhang et al. 2019). Interestingly, the negative effect of a reduction in rainfall on fine root biomass has been shown to decline over time (Zhang et al. 2019), which suggests that trees slowly acclimate to a changing precipitation regime.

Plasticity of the root system can be a strategy to acclimate to climate change-induced shifts in resource availability (Nicotra et al. 2010). Tree roots have been observed to develop plastic responses to different physical and chemical soil conditions (Weemstra et al. 2017; Zadworny et al. 2017). This plastic response of the root phenotype includes changes in the spatial arrangement or distribution of the root system as a whole, i.e., fine root mass by depth in response to soil types and/or water availability in different soil depth layers (Bardgett et al. 2014; Weemstra et al. 2017). Despite plasticity being a critical short term acclimation response of temperate and boreal tree species to climate change induced drought (Aubin et al. 2016), it has largely been ignored in previous studies (Shipley et al. 2016; Valladares et al. 2014; Violle et al. 2012).

A predictor of root plasticity in response to the environment might be species growth in terms of aboveground woody biomass, as previous studies have indicated that selective root placement might be positively correlated with fast growth of plants (Bardgett et al. 2014; Grime & Mackey 2002; Kembel et al. 2008). The more selective placement of fine roots could potentially lead to greater fine root biomass plasticity for faster growing plants under varying levels of water availability. In support, higher root biomass plasticity of faster growing plants has been found for trees in the temperate forest, (Maseda & Fernández 2016; Rytter 2013; Zwetsloot & Bauerle 2021), the Mediterranean forest (Mayoral et al. 2016) and for grapevines (Bauerle et al. 2008). On the contrary, a negative relationship between fast growth rate and plasticity in root biomass in response to irrigation and fertilization has been found in tropical forests species (Noulèkoun et al. 2017). The positive correlation of plasticity in resource acquisition in response to different environmental conditions and plant growth rate calls for further investigation (Bardgett et al. 2014).

The main objective of our study was to examine how fine-root acclimation capacity in terms of absorptive fine root biomass and rooting depth varied among *Betula papyrifera*, *Quercus rubra*, *Acer saccharum*, *Larix laricina*, *Pinus strobus* and *Picea glauca*, exposed to high or low growing season water availability. Furthermore, we aimed to show how absolute absorptive fine root density (i.e., the three most distal root orders in mg cm^−3^) responded to high and low water availability depending on species and soil depth layers. Based on previous findings by Zhang et al. (2019), we hypothesized that trees exhibit lower absorptive fine root density under low growing season water availability. Given the evidence of fine root plasticity response to water (Bauerle et al. 2008; Maseda & Fernández 2016; Mayoral et al. 2016; Rytter 2013; Takenaka et al. 2016; Ye et al. 2019; Zwetsloot & Bauerle 2021), we hypothesized that early successional, fast growing tree species would exhibit higher plasticity in absorptive fine-root biomass and rooting depth given varying water availability than late successional, slower growing tree species.

## Material & methods

### Study site

This study was conducted at the IDENT-SSM experimental site established in 2013 near Sault Ste. Marie, Ontario, Canada (46.546610° N, -84.455650° W, 220 m a.s.l.) (Belluau et al. 2021). The experimental site is characterized by a humid and continental climate with a mean annual precipitation of 898 mm and a mean annual temperature of 4.7 °C (Belluau et al. 2021). Climatic moisture deficits (potential evapotranspiration > precipitation) from May through August are common (Belluau et al. 2021). The soil is classified as an Eluviated Dystric Brunisol, and is relatively infertile, rapidly drained, and sandy loam to loamy sand in texture with a pH of 5.2 (Belluau et al. 2021).

This trial consists of six native tree species planted in the year 2013: *Acer saccharum* Marsh. (As), *Betula papyrifera* Marsh. (Bp), *Larix laricina* K. Koch (Ll), *Pinus strobus* L. (Ps), *Picea glauca* (Moench) Voss (Pg) and *Quercus rubra* L. (Qr). These species were selected for their broad range in shade tolerance for both the gymnosperm (Pg > Ps > Ll) and angiosperm (As > Qr > Bp) species (Belluau et al. 2021). Four replicate blocks of monocultures (*n* = 6) were exposed to a high or low growing season (June – August) water treatment beginning in spring 2014. The high water treatment was implemented through weekly irrigation of 45 mm of precipitation from June 1^st^ to August 31^st^, while the low water treatment was created by a 25% rainfall exclusion apparatus (Belluau et al. 2021). The irrigation treatment increased the amount of water received during this period by more than 250% of normal growing season precipitation, and therefore created saturated soil conditions. Belluau et al. (2021) provide further details regarding experimental design and treatment.

### Root sampling

Roots were collected from the upper 30 cm of soil in each of the 48 monoculture plots from August 15^th^ to September 14^th^, 2018, the sixth growing season. This seasonal period of sampling was selected to coincide with maximum root production of temperate tree species (Burke & Raynal 1994). Three soil cores of 5.08 cm in diameter were collected to a depth of 30 cm along a systematic, diagonal transect in the central area of each plot. Diagonal transects ran from the north-eastern corner to the south-western corner of each plot. Cores were taken at the midpoint between a 2 × 2 cluster of seedlings to maximize the amount of root tissue collected from all individual trees and avoid bias by one tree. If dead trees were present in the cluster at the desired location, sampling was shifted to the next available cluster midpoint. Cores were collected using a Signature Rubber Coated Slide Hammer and a 2″ × 12″ Split Soil Corer SCS w/Core Tip with a cross handle (Arts Machine Shop Inc., American Falls, ID, USA). The split steel cylinder allowed the sectioning of five discrete soil strata (0–5, 5–10, 10–15, 15–20 and 20–30 cm) using a sharpened implement to carefully sever the roots. In total, 144 separate cores were collected and separated into 720 individual strata. Root samples were transported in a cooler and stored within 24 h in a freezer at -25 °C for a maximum period of 4 months until processing. Samples were thawed and pooled by plot and depth, and roots were washed gently free of soil using a GVF Hydropneumatic Root Washer (Gillison’s Variety Fabrication, Inc., Benzonia, MI, USA) and an 840 µm nylon sieve to capture any loose roots. To minimize loss of root tissue, the piping of this apparatus was manually flushed between each sample to collect roots not retrieved by sieving.

Roots were hand sorted in a tray of water into five different fractions using the functional classification approach (Freschet & Roumet 2017). The five fractions were absorptive fine roots (first three root orders), transportive fine roots (fourth root order), coarse roots (> 2 mm diameter), dead roots, and fine root fragments. The dead fine roots were identified by their greater brittleness, darker color, and the lower cohesion between the cortex and periderm tissues. Fine root fragments can account for a significant amount of total fine root biomass (Bauhus & Bartsch 1996). Fine roots were classified as fragments if they belonged to the absorptive fine root category and were < 1 cm in length (Lei et al. 2012). Fragments were picked from marked boxes, randomly distributed in the sorting tray and represented 10% of the tray surface area. Total biomass of fine root fragments was multiplied by 10 and allocated to the absorptive fine and dead root fractions in proportion to their amount in a given sample (Lei et al. 2012). All root fractions were dried at 65 °C for 72 h to determine dry mass. The skeletal volume of soil in each sample was measured, applying the principle of Archimedes (Hughes 2005). The density of each root fraction was calculated as the ratio of root dry mass to soil volume.

The weighted mean rooting depth, i.e., the vertical center of gravity of absorptive fine roots for each tree species, plot and water availability treatment was estimated as

$$Weighted~mean~depth=\frac{\left(\left({M}_{0-5cm}\times 2.5cm\right)+\left({M}_{5-10cm}\times 7.5cm\right)+\cdots +\left({M}_{20-30cm}\times 25cm\right)\right)}{{M}_{0-30cm}}$$

where M is the mass of the absorptive fine roots in a given soil layer weighted by the center of that layer (Archambault et al. 2019).

### Plasticity

Plasticity of the absorptive fine root biomass and rooting depth was estimated using four different indices allowing contrasting evolutionary and ecological indicators to be interpreted (Valladares et al. 2006). Furthermore, the use of more than one index facilitates the comparison to other studies and future meta-analyses (Valladares et al. 2006). The total phenotypic variability of each species was estimated by the coefficient of variation (CV) for the entire data set, i.e., standard deviation divided by the mean (Tobner et al. 2013). The CV is useful for exploring phenotypic variability in general, including developmental instability (Valladares et al. 2006). The coefficient of variation for data pooled over the two water treatments (CVm) was estimated as the standard deviation of mean root density divided by the mean of the root density water treatment means. This index removes the weak point of mixed variability within and between environments as is the case with CV (Valladares et al. 2006). The plasticity (PI) of each species was calculated as:

PI = [maximum (mean root density between water treatments) – minimum (mean root density between water treatments)] / maximum [mean root density between water treatments].

The PI is the most robust, simple, and widely used of our four indices of plasticity (Valladares et al. 2006). The proportion of total phenotypic variation in root density due to differences in water treatment (PI:CV) was calculated as the ratio of PI to CV (Tobner et al. 2013). Phenotypic variability due entirely to acclimation to water treatment was indicated by a PI:CV value of 1 (Tobner et al. 2013).

Total aboveground woody biomass after six years of growth was used as a proxy of species growth rate and resource use strategy. Woody biomass for each species and plot was estimated using site-specific allometric equations derived from destructive sampling of selected block level buffer trees (Belluau et al. 2021).

### Statistical analysis

All statistical analyses were carried out using the R software (R Core Team 2021). Significance threshold was set to *p* = 0.05. The data was analyzed using linear mixed-effect (LMM) and generalized linear mixed-effect models (GLMM) with the *lmer* and *glmer* function from the R package _LME_4 (Bates et al. 2015). Type-III analysis-of-variance (ANOVA) was conducted with the R package _CAR_ (Fox 1997), and _LMERTEST_ (Kuznetsova et al. 2017). Despite the absence of a significant water availability effect in the full model, *post-hoc* tests allowed us to evaluate pairwise comparisons between fixed effects given the significant tree species and soil depth results. To compare means of single fixed effects and their interactions*, post-hoc* analysis of each model was performed with the *emmeans* function from the R package _EMMEANS_ (Tukey’s Honest Significant Difference Test) (Lenth 2021).

Prior to model development, collinearity was assessed by producing a matrix of scatter plots. Only non-collinear (r < 0.5) variables were included in the model. A visual based approach was applied to perform model diagnostics by checking model residuals against predicted values.

We tested our first hypothesis with a LMM to quantify the effect of the predictor’s species, soil depth and water treatment on the log-transformed (x + 1) response variable absorptive fine root density. We defined root density as milligrams per cubic centimeter of soil (mg cm^−3^). The model included all 2- and 3-way interactions of the three fixed factors (tree species, soil depth and water treatment). Block and plot were included in the models as random factors with all the fixed factor interactions to account for spatial heterogeneity and dependence among soil depth layers among plots (Barr et al. 2013; Clark & Linzer 2015). To reduce the variance within treatment blocks and maximize variance among blocks we paired each high water treatment block with a low water treatment block (block 1 + 2, 3 + 4, 5 + 8, 6 + 7). A box-cox transformation was performed on weighted mean rooting depth (cm) to achieve normality, and a LMM was applied testing tree species and water treatment as fixed factors and block as a random factor. The random term “plot” was included in all previous models but was dropped from the model for weighted mean rooting depth since accounting for dependence among soil depth layers was not needed. Dead root density (mg cm^−3^) could not be normalized with a transformation. Hence, each value of dead root density was multiplied by ten and rounded to the nearest integer to apply a GLMM with a Poisson distribution, the optimizer “bobyqa” and 60,000 iterations (Bates et al. 2015) using the same model structure as for fine root density.

We tested our second hypothesis by bootstrapping (i.e., random resampling with replacement) applied before and after calculating plasticity indices for each species (six individual values) to examine the relationship of plasticity with mean aboveground woody biomass across water treatments. Species populations were created with a total of 6000 iterations (*N* = 1000 per species). The bootstrap data was then analyzed with linear regression models (LM’s) to test for the effect of aboveground woody biomass on plasticity. Multiple *R*^2^ values from the linear models were obtained with the *summary* R function.

## Results

### Absorptive fine root density

**Fig. 1:**
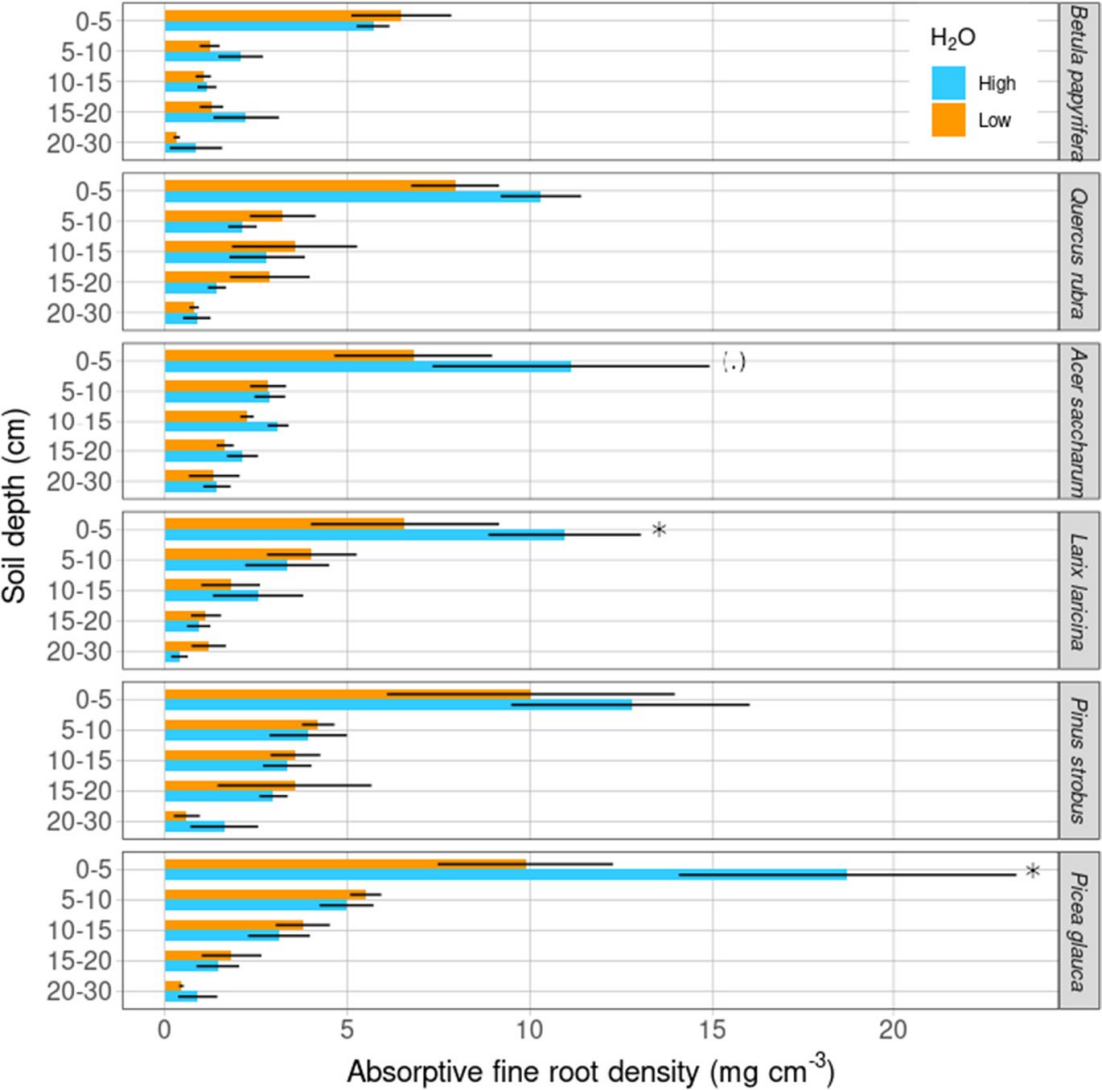
Variation in mean (± standard error of the mean) absorptive fine root density with soil depth for six tree species in the high (blue) and low (orange) water treatments (H_2_O). Significant water treatment effects for a given soil depth are noted by an asterisk and marginal effects by a dot (‘*’, *p* < 0.05 — * p* < 0.1 ‘.’). From top to bottom, data are presented in order of increasing shade tolerance within broadleaved and conifer groups respectively

Total absorptive fine root density depended strongly on tree species (Table 1, *F* = 4.90, *p* < 0.01). Across the entire 0–30 cm depth, absorptive fine root density ranged from a minimum of 0.96 mg cm^−3^ for *Larix laricina* under low water availability to a maximum of 8.14 mg cm^−3^ for *Picea glauca* under high water availability. Furthermore, total absorptive fine root density declined strongly with increasing soil depth showing a double to triple order of magnitude difference, from 9.7 ± 0.8 mg cm^−3^ at 0–5 cm to 0.9 ± 0.1 mg cm^−3^ at 20–30 cm across all tree species (Table 1, Fig. 1, *F* = 113.58, *p* < 0.001). Despite absorptive fine root density not showing a significant difference between the high and low water treatment across tree species and soil depth layers (Table 1), there was a marginal soil depth by water availability interaction (Table 1, *p* = 0.06) and *post-hoc* tests revealed differences in absorptive fine root density in response to water availability when looking at individual tree species in the 0–5 cm depth layer (Table 1, Fig. 1; Table S1). Absorptive fine root density decreased by -40% for *Larix laricina* (Table S1, *p* < 0.01), and by -46% for *Picea glauca* (Table S1, *p* < 0.05) from high to low water availability in the 0–5 cm soil depth. *Acer saccharum* showed a marginally significant decrease of -39% between high and low water availability in the same soil depth (Table S1, *p* = 0.09). Averaged over all species, soil depth layers, and water treatments, mean absorptive fine root density was 3.7 ± 0.2 mg cm^−3^.

**Table 1:** Results of Type III analyses of variance testing for the effects of tree species (*Betula papyrifera*, *Quercus rubra*, *Acer saccharum*, *Larix laricina*, *Pinus strobus*, and *Picea glauca*), soil depth (cm), and high and low water (H_2_O) treatment on absorptive fine root density, dead fine root density, and weighted mean rooting depth (cm). The *X*^2^ values for dead fine root density result from the Poisson distribution

Source of variation	Effect on absorptive fine root density (mg cm^−3^)			Effect on dead fine root density (mg cm^−3^)		Effect on weighted mean rooting depth (cm)	
	DF	*F*	P	*X*^2^	P	*F*	P
Species	5	4.90	** < 0.01**	141.80	** < 0.001**	1.38	0.25
Soil depth	4	113.58	** < 0.001**	37.22	** < 0.001**	-	-
H_2_O	1	1.45	0.31	2.86	**0.09**	2.35	0.13
Species × soil depth	20	1.58	0.06	196.64	** < 0.001**	-	-
Species × H_2_O	5	0.36	0.86	8.79	0.11	0.93	0.47
Soil depth × H_2_O	4	2.28	0.06	16.18	** < 0.01**	-	-
Species × soil depth × H_2_O	20	0.70	0.81	73.67	** < 0.001**	-	-

### Dead root density

**Fig. 2:**
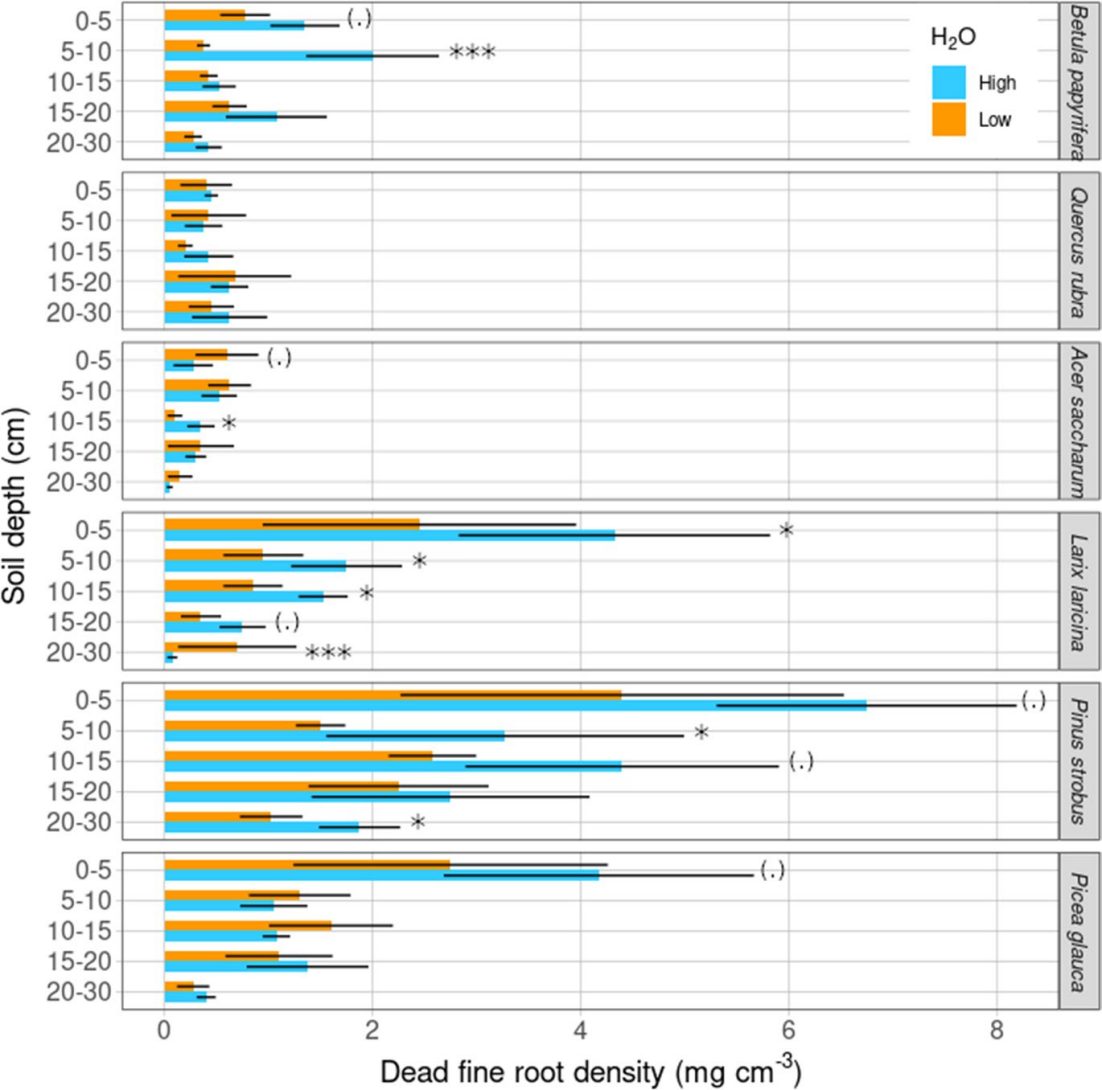
Variation in mean (± standard error of the mean) dead fine root density with soil depth for six tree species in the high (blue) and low (orange) water treatments (H_2_O). Significant water treatment effects for a given soil depth are noted by an asterisk and marginal effects by a dot (‘*’, *p* < 0.05—*p* < 0.1 ‘.’). From top to bottom, data are presented in order of increasing shade tolerance within broadleaved and conifer groups respectively

Although dead fine root density varied more strongly in response to tree species (Table 1, *X*^2^ = 141, *p* < 0.001) and soil depth (*X*^2^ = 37, *p* < 0.001), than to the water treatment (*X*^2^ = 2.8, *p* = 0.09), the interaction between tree species, soil depth, and water treatment indicated that mortality rates of fine roots depended on the species specific response to the water treatment per soil depth layer (Table 1, *X*^2^ = 73, *p* < 0.001). Averaged over all tree species and water treatments, dead fine root density generally declined by 78% from the upper soil layer (0–5 cm) to the bottom layer (20–30 cm) (Fig. 2). *Post-hoc* test results revealed that *Larix laricina* showed a -100% decrease of dead fine root density in the 20–30 cm depth interval under high water availability (Table S2, Fig. 2, *p* < 0.001) while the decrease of *Acer saccharum* was marginal (Table S2, Fig. 2, *p* = 0.08). Angiosperms (*Betula papyrifera*, *Quercus rubra*, and *Acer saccharum*) did not reduce their dead fine root density with soil depth to the same extent as gymnosperms (*Larix larcina*, *Pinus strobus*, and *Picea glauca*), (Table 1, Fig. 2). The pooled mean dead root density of the three conifer species was 1.46 mg cm^−3^, 65% higher than that of the three broadleaved species, suggesting faster decomposition of angiosperm fine roots (Fig. 2). The effects of the water treatment on dead root density tended to be greater in upper soil layers, as opposed to the deeper soil layers (Table 1, Fig. 2).

### Mean rooting depth of absorptive fine roots

**Fig. 3:**
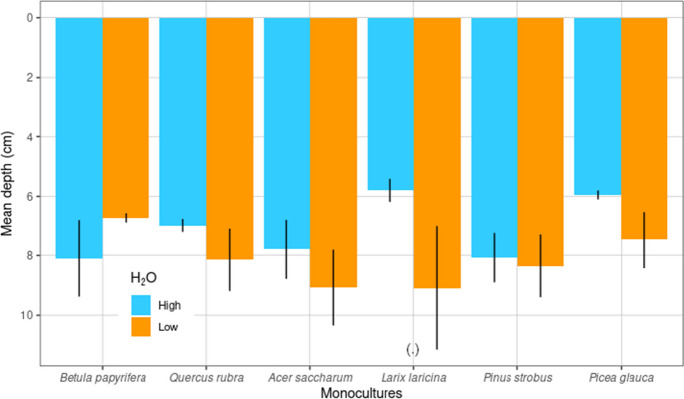
Variation in weighted mean rooting depth of absorptive fine roots in cm (± standard error of the mean) for six tree species in the high (blue) and low (orange) water treatments (H_2_O). Marginally significant water treatment effects for a given soil depth are noted by a dot (*p* < 0.1 ‘.’). From left to right, data for broadleaved and conifer species are presented separately in order of increasing shade tolerance

Although none of the tree species showed significant changes in weighted mean rooting depth (i.e., the vertical center of gravity of the fine roots) in response to the water availability treatment (Table 1, *F* = 0.93, *p* = 0.47), we observed some trends of increased rooting depth under low water availability (Table 1, Fig. 3). *Larix laricina* showed a marginally significant increase in mean rooting depth of 57% in response to low water availability (Table S3, Fig. 3, *p* = 0.06). Mean rooting depth of absorptive fine roots tended to increase by 8% in the low water treatment for all species except *Betula papyrifera* where the opposite trend was observed (Fig. 3, Table S3). Weighted mean rooting depth ranged from a minimum of 4.5 cm under high water availability to a maximum of 14.6 cm under low water availability for *Larix laricina*. Across all species the mean depth of absorptive fine roots was 7.7 cm (± 0.3).

### Plasticity of absorptive fine root biomass in the 0–5 cm soil depth and of weighted mean rooting depth (0–30 cm soil depth)

**Fig. 4:**
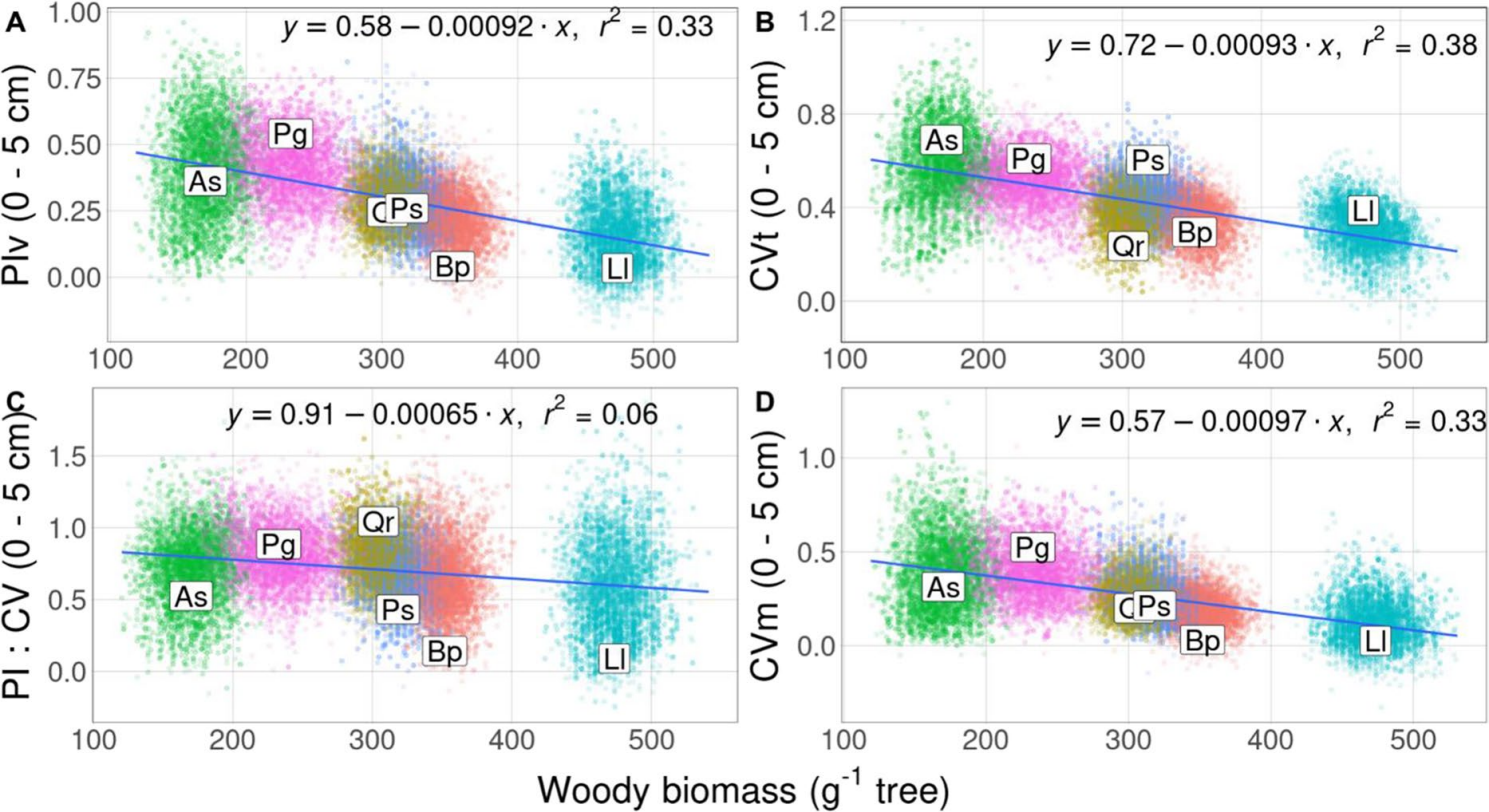
Relationship between plasticity of absorptive fine root biomass in the upper soil layer (0–5 cm) and aboveground woody biomass (A: PIv = (Maximum mean-minimum mean)/maximum mean; B: CVt = Standard deviation/mean; C: PI:CV = PIv/CVt; D: CVm = Standard deviation of means/mean of means). The linear regression model for each relationship and the regression line with a 95% confidence interval are shown. Species centroids (original, individual plasticity values) are shown by abbreviations Bp: *Betula papyrifera* (orange), Qr: *Quercus rubra* (yellow), As: *Acer saccharum* (green), Ll: *Larix laricina* (turquoise), Ps: *Pinus strobus* (blue), Pg: *Picea glauca* (purple)

**Fig. 5:**
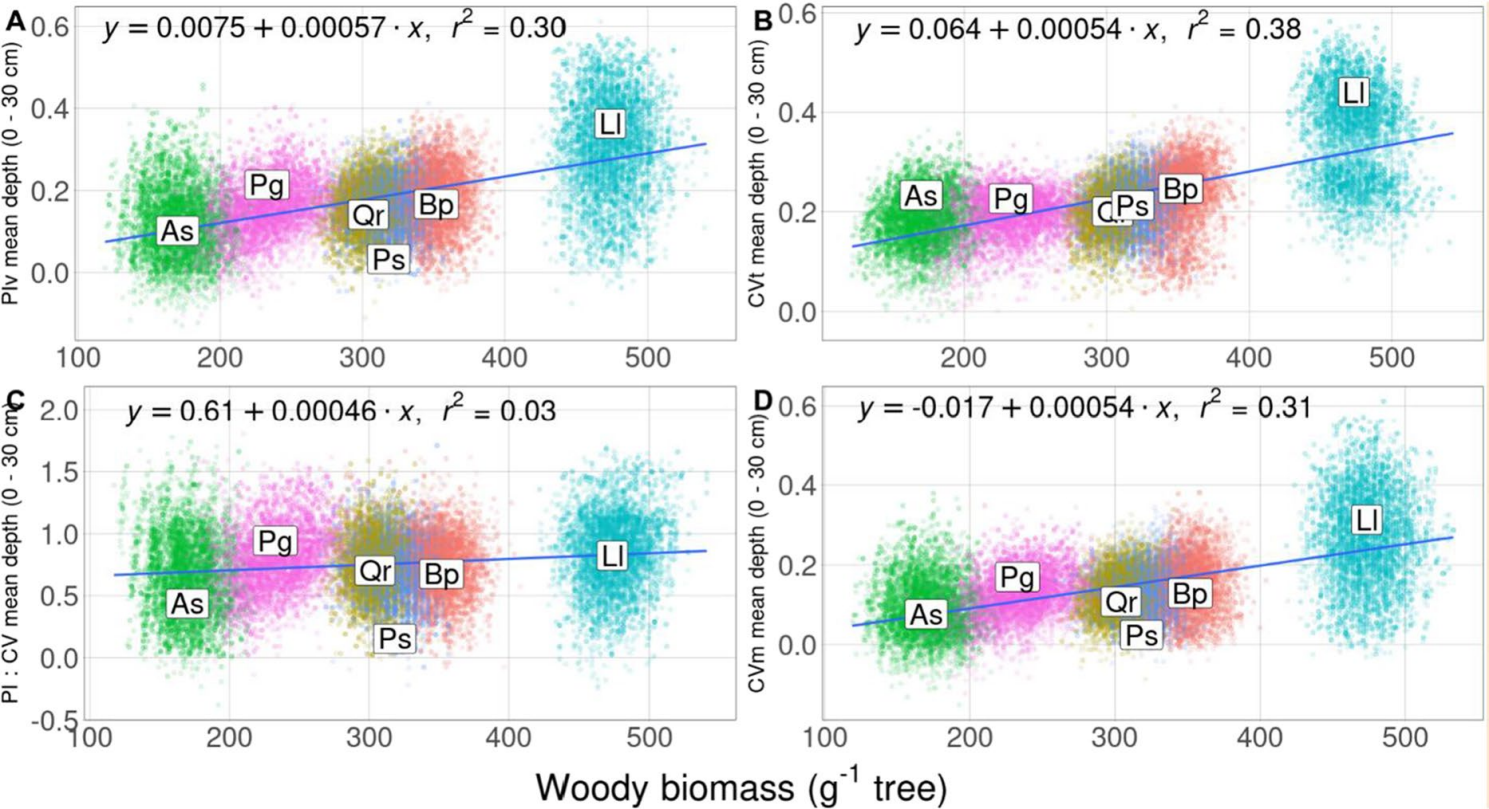
Relationship between plasticity of mean rooting depth and aboveground woody biomass (A: PIv = (Maximum mean-minimum mean)/maximum mean; B: CVt = Standard deviation/mean; C: PI:CV = PIv/CVt; D: CVm = Standard deviation of means/mean of means). The linear regression model for each relationship and the regression line with a 95% confidence interval are shown. Species centroids (original, individual plasticity values) are shown by abbreviations (Bp: *Betula papyrifera* (orange), Qr: *Quercus rubra* (yellow), As: *Acer saccharum* (green), Ll: *Larix laricina* (turquoise), Ps: *Pinus strobus* (blue), Pg: *Picea glauca* (purple)

All four plasticity indices showed a significant negative linear relationship between absorptive fine root biomass in the upper soil layer (0–5 cm) and total aboveground woody biomass in year 6 (Fig. 4, Table S4, *p* < 0.001). Plasticity in absorptive fine root biomass in the 0–5 cm layer tended to be higher with lower aboveground woody biomass as a proxy for successional status of tree species in all four indices (Fig. 4). Total phenotypic variability, PIv and CVm exhibited stronger negative relationships with total aboveground woody biomass than PI:CV (Fig. 4, Table S4). In contrast, weighted mean rooting depth exhibited a significant positive linear relationship with total aboveground woody biomass for all four plasticity indices (Fig. 5, Table S5, *p* < 0.001). Similar to absorptive fine root biomass, the PI:CV displayed a weaker linear relationship with total aboveground woody biomass than CV, PIv and CVm (Fig. 5, Table S5).

## Discussion

### Absorptive fine root density

Contrary to our general hypothesis that absorptive fine root density would be reduced under low water availability, this pattern was not observed for most of our species and soil depth layers (Fig. 1, Table 1 & S1). However, two of the six temperate tree species showed some ability to acclimate in terms of absorptive fine root response to the water treatment with increased absorptive fine root density for *Larix laricina* and *Picea glauca* in the 0–5 cm soil depth under high water availability (Fig. 1, Table S1), showing some support for our hypothesis and in agreement with previous meta-analyses (P. Wang et al. 2020; Zhang et al. 2019). Although the absorptive fine root density response to water availability was weaker than expected, there was a clear trend towards greater absorptive fine root density in the upper 0–5 cm as opposed to deeper layers under the high water treatment, except for *Betula papyrifera* (Fig. 1). This trend for most tree species to increase absorptive fine root density in the upper soil layer under the high water treatment could be explained by the greater availability of water (and consequently nutrients) in the upper soil layer for foraging roots (Fig. 1). In support of this interpretation, Maxwell et al. (2020) identified water availability as a driving factor of soil nutrient availability in the topsoil on another site of the international Tree Diversity Network (TreeDivNet) in France. It remains a limitation of this study that we did not measure absorptive fine root turnover. Increased root respiration and turnover under drought can lead to more efficient water uptake from younger roots (Brunner et al. 2015). These younger roots that appear lighter in terms of dry weight in our study could show higher absorptive capacity.

As trees can increase their absorptive fine root surfaces in response to differences in water availability within the same fine root dry weight, assessing the fine root density response alone can only give an “incomplete” picture of the overall fine root response. It is rather the reduction of absorptive fine root density alongside an increase in, for example, absorptive fine root branching density (number of root tips per centimeter root length) under low water, which might be a cheaper strategy in terms of carbon investment for the trees to maintain absorptive capacity (Jaeger et al. n.d.). However, absorptive fine root density and rooting depth plasticity play an important role in the tree’s response to differences in soil water availability (Fig. 4 & 5).

Since soil clay content can have a positive effect on soil moisture (Borden et al. 2020), and sandy soils generally have a lower capacity of soil water retention (Hillel 2003), the coarser soil texture with increasing soil depth on our research site could have made the upper soil layers more favorable for fine root foraging. For example, Weemstra et al. (2017) showed that *Fagus sylvatica* and *Picea abies* showed three times higher fine root biomass in the upper soil layer on sandy soils compared to clay soils, and *F. sylvatica* partitioned two-thirds of its fine root biomass to the organic and upper (0–30 cm) mineral soil layer in medium- to coarse-grained meltwater sands (Meier et al. 2018). Comparing the percentage of root biomass in the organic soil layer on silicate-rich acid Triassic sandstone and silicate-poor acid Pleistocene sandy deposits, Hertel et al. (2013) showed that a greater percentage of *F. sylvatica* root biomass was partitioned to the organic layer on sandy sites. However, these pronounced differences between top- and subsoil fine root densities of *Fagus sylvatica* have been found in a broad range of different soil textures from sandy loam to silt (Kirfel et al. 2019). Similarly, on sandy podzol *Betula pendula* developed more roots in the topsoil under irrigation (Altinalmazis-Kondylis et al. 2020). Furthermore, close to our research site *Larix laricina* and *Picea mariana* demonstrated a shallow root habit on a sandy plain (Bannan 1940), and *Pinus sylvestris* partitioned most of its fine root biomass to the organic soil horizon on sandier soil compared to fine-textured soil along a chronosequence of soil development (Fujii et al. 2021). This tendency of trees to partition most of their root biomass into the upper soil and/or organic layer could be caused by the opportunistic growth of tree roots towards the part of the soil column where they can encounter most of the water and nutrients (Zanetti et al. 2015).

One could argue that the higher absorptive fine root densities we measured under the high water treatment might simply be the result of above- and belowground allometric scaling effects, because trees grew larger under more benign growing conditions. However, absorptive fine root density ratio (absorptive fine root density per soil depth layer compared to total fine root density in the entire soil column, Fig. S1), dead fine root density ratio (Fig. S2), root to shoot ratio (Fig. S3 & Table S6, S7), leaf biomass to absorptive fine root ratio (Fig. S4 & Table S8, S9) and woody biomass to root ratio (Fig. S5 & Table S10, S11) were not significantly altered by the water treatment. Furthermore, aboveground woody biomass was not significantly affected by our water treatment except for *Larix laricina* (Table S12 & S13).

It remains important to acknowledge that we studied young and relatively small trees, and that the response of older and larger trees to differences in soil water availability could be different. Age/size can act as a mortality trigger under drought, while on the other hand older/larger trees can develop a stress memory to repeated droughts and their tolerance might be increased through larger accumulation of non-structural carbon pools (Allen et al. 2015; Amaral et al. 2020; Niinemets 2010). Furthermore, older/larger trees might be less plastic in their fine root biomass response to differences in water availability (more conservative), while younger/smaller trees are better able to acclimate (more acquisitive) (Li et al. 2021; Niinemets 2010). In support, young/small *poplar* showed increased fine root biomass under drought, while older/larger poplar showed a decrease (Geng et al. 2022). However, a rapid re-allocation of carbon in the rhizosphere of 100-y-old *Scots pine* under drought has also been reported (Joseph et al. 2020). Although we did not detect significant differences in above versus belowground response to high and low water (Fig. S3), it is important to acknowledge that older/larger trees would have needed to support a much larger above ground biomass with possibly only slightly increased fine root biomass/density (Helmisaari 1995). Further research is needed to assess the tree age/size dependent response to drought (Bréda et al. 2006).

It is often the simultaneous stresses exerted by both press- (ongoing climate change) and pulse-drought events which cause the greatest disturbance in ecosystems (Harris et al. 2018). Our study focused on press drought which remains important, as press-droughts lead to a higher frequency of persistent forest decline and can delay recovery more than pulse-droughts (Jiao et al. 2021). As water availability typically depends on unpredictable precipitation events, plants do not respond very strongly to relatively short drought pulses (Poorter et al. 2012). Under press drought, stronger root growth of slower growing vines (Bauerle et al. 2008) and decreased *sugar beet* root dry weight was observed (Fitters et al. 2018). On the other hand, Padilla et al. (2013) found no difference in temperate grasslands root mass between pulse and press watering. The combined and separate effects of climate change induced press and pulse water availability on fine roots deserves further research and remains a limitation of our study.

Like Archambault et al. (2019), evergreeness was a strong driver of absorptive fine root density in our study. Archambault et al. (2019) collected their root samples from another IDENT site situated in the temperate forest of North America, and evergreens allocated proportionally more biomass to belowground roots than aboveground parts in mixtures and monocultures. At least in the early years of tree growth, Archambault et al. (2019) concluded that deciduous trees might be more efficient in absorbing soil resources than evergreens, and hence need to invest less biomass into roots. Few studies have investigated the relationship between evergreeness and fine root density and/or biomass. In a study from China, 10-year-old *Pinus tabulaeformis* showed four to five times higher fine root biomass than *Robinia pseudoacacia* (Chen et al. 2016), while a study from the European temperate forest identified the abundance of conifers as driver of fine root biomass in the organic, but not the mineral soil layer (Finér et al. 2017). Similarly, evergreens produced more roots than broadleaves in an urban environment (Lu et al. 2021), and higher fine root production was also measured in monocultures and mixtures of *Picea abies* and *Pseudotsuga menziesii* compared to *Fagus sylvatica* and *Quercus petraea* (Lei et al. 2012). Contrary to our results, higher fine root biomass has been found in *Fagus sylvatica* compared to *Picea abies* and *Pinus sylvestris* (Finér et al. 2007), and *beech* has been shown to partition more fine root biomass into the mineral soil than *Pinus sylvestris* (Förster et al. 2021). Furthermore, global studies investigating the relationship of above- and belowground biomass allocation determined that: (i) gymnosperms invested more biomass into leaves than angiosperms (Poorter et al. 2015), (ii) there was no difference between the root to shoot ratio of angio- and gymnosperms (Ledo et al. 2017; Mokany et al. 2006), and (iii) there was an increase of root biomass to foliar biomass under colder climate conditions in both angio- and gymnosperms (Reich et al. 2014). An explanation for the higher fine root biomass of evergreens could be a lower turnover rate of roots, which would lead to a higher fine root biomass accumulation at any given point in time (McCormack et al. 2014; See et al. 2019). However, this explanation is not supported by previous studies, since no clear differences in fine root turnover rate have been found between deciduous and coniferous species (Augusto et al. 2015; Brunner et al. 2013). Furthermore, total root production and turnover rate have been positively linked (McCormack et al. 2014). Archambault et al. (2019) proposed that since evergreen tree species have evolved to grow on poorer soils, they might be less plastic and unable to adapt to more benign growing conditions, and hence not able to reduce their fine root biomass to the same extent as broadleaves. However, we did not detect lower fine root biomass plasticity for evergreen trees in our study. Therefore, it could simply be that evergreens follow a more conservative strategy when growing their roots and keep generally higher fine root biomass stocks to be prepared for eventual drought periods. In support of this interpretation, in a drought simulation experiment *Juniperus monosperma* and *Pinus edulis* shifted their water uptake among existing roots rather than growing new roots (Mackay et al. 2019). Measures of fine root production, turnover and biotic interactions would be needed to better explore the relationship of clade and root biomass.

### Mean rooting depth under high and low water availability

The only marginally significant increase of mean rooting depth observed under low water availability for *Larix laricina* might be explained by the prevailing climatic (high rainfall) and site conditions. The study site is located on a lower foot slope with good drainage and frequent top-wetting of the soil by naturally occurring precipitation, that might reduce the need for the fine roots to grow much deeper (Fan et al. 2017) (Fig. 3). Our results contrast with previous meta-analyses which determined an increase of rooting depth under reduced water availability conditions (Brunner et al. 2015; Zhang et al. 2019). However, it must be noted that our water treatment was not comparable to a drought treatment in terms of severity of water scarcity.

Another possible reason why species weighted mean rooting depth did not increase significantly in response to the water treatment was the presence of a plough layer at 25 cm soil depth on our research site, formerly managed for low-input agriculture (personal communication). Repeated ploughing can develop a soil hardpan underneath, which hinders the free movement of water and the penetration of roots into deeper soil layers (Amanullah et al. 2010). Furthermore, a plough pan can lead to greater reduction of soil hydraulic conductivity with increasing soil depth (Wencai et al. 2019), and soil water holding capacity can remain lowered for a long period of time (Brudvig et al. 2013). Even distant past land use legacies can influence the present day physical and chemical soil properties (Blondeel et al. 2019; Nikodemus et al. 2022; Verheyen et al. 1999).

### Dead root density under high and low water availability

The higher dead root density we observed under high water availability, especially for evergreens, could be driven by generally higher fine root production and more recalcitrant fine roots, and hence slower fine root decomposition rates (Augusto et al. 2015) (Fig. 2). However, this interpretation is not supported by two meta-analyses by C. Wang et al. (2018) who found an increase of fine root necromass (biomass/necromass ratio) with decreasing mean annual precipitation, and a higher fine root necromass for angiosperms in the temperate forest (C. Wang et al. 2022). Similarly, two studies which manipulated rainfall by roof exclusion measured higher fine root mortality and necromass under low water availability while fine root biomass was generally higher under normal precipitation (control) (Konôpka et al. 2007; Persson et al. 1995). Studies utilizing increasing aridity along time series also found a general trend of higher necromass with drier environments (Eissenstat et al. 2000; Konôpka 2009; Konôpka & Lukac 2013; Makkonen & Helmisaari 1998; Montagnoli et al. 2019), and support for this trend also comes from studies along precipitation gradients (Fuchs et al. 2020; Meier & Leuschner 2008). Interestingly, *Picea abies* increased root suberization under drought conditions indicating a ‘slow’ ecological strategy, while *Fagus sylvatica* increased the production of thin, ephemeral, absorptive fine roots during drought indicating a ‘fast’ strategy (Nikolova et al. 2020). Increased fine root suberization acts as a mechanism to reduce water loss from the roots to the drying soil, but can also prevent the free flow of water and nutrients through the root and therefore reduce hydraulic conductivity (Steudle 2001). Our results are partially supported by studies that did not find a strong necromass response to increasing or decreasing precipitation (Fuchs et al. 2020; Leuschner et al. 2001, 2004). Partial support also comes from a root litter decomposition study, where *P. sylvestris* root litter decomposed slower in terms of root mass loss than *C. betulus* although this only occurred in standardized litter as opposed to site-specific litter (Wambsganss et al. 2021). Contrary to our results, lower fine root biomass, necromass, productivity, and turnover have been found for *Pinus sylvestris* compared to *Fagus sylvatica* (Förster et al. 2021). It remains a limitation of our study that we did not measure fine root turnover, and decomposition rates, as such measures would have facilitated the interpretation of our dead fine root density results.

### Fine-root plasticity response to high and low water availability

Our results partially support our second hypothesis suggesting that early successional, fast growing tree species would exhibit higher plasticity in absorptive fine roots to increased and decreased water availability. However, late successional species such as *Acer saccharum* and *Picea glauca*, characterized by slow aboveground woody biomass growth, showed higher plasticity in fine root biomass allocation in the topsoil. In contrast, early successional species, such as *Betula papyrifera* and *Larix laricina*, showed higher plasticity in rooting depth (0–30 cm) (Fig. 4 & 5). Absorptive fine root plasticity might explain why a previous study from our research site found that four out of six tree species showed little response to the high and low water treatment in terms of aboveground growth (Belluau et al. 2021). This absence of aboveground growth response could be related to different fine root acclimation potential that tends to vary with tree species successional status (Fig. 4 & 5). Support for this interpretation comes from the observation that overall aboveground woody biomass did not change significantly in response to the water treatment (Table S12 & S13). Our results concur with higher fine root plasticity in faster growing trees (Altinalmazis-Kondylis et al. 2020; Takenaka et al. 2016; Ye et al. 2019), vines (Bauerle et al. 2008) and grasses (Hanslin et al. 2019). Furthermore, late successional *beech* has been reported to have less plasticity in vertical fine root biomass allocation pattern in response to changes in water availability (Leuschner 2020) and has been observed to show very little change in vertical fine root allocation even under severe drought conditions (Mainiero & Kazda 2006). As fast growing, early successional tree species need more water to satisfy high transpiration rates, they might rely more heavily on deep soil water exploration during periods of water scarcity (Flo et al. 2021), which could lead to a greater potential for rooting depth plasticity.

Shallow versus deeper root foraging of late and early successional tree species through absorptive fine root plasticity could be related to the species rooting habit. *Acer saccharum* rooting habit has been described as having large, lateral roots spreading horizontally outward (Biswell 1935), while *Picea glauca* rooting habit has abundant, shallow, laterally spreading roots with sinkers (Bannan 1940; Eis 1970; Jeffrey 1959; Strong & La Roi 1983). In contrast, early successional *Quercus rubra* and *Pinus strobus* exhibit a clear taproot (Duncan 1941; Emerson 1921; Holch 1931; Pulling 1918). The ability of species with a taproot such as *Quercus rubra* and *Pinus strobus* to reach deeper soil layers (Burns & Honkala 1990; Duncan 1941; Holch 1931) might have made it unnecessary for these species to develop plastic absorptive fine roots in the upper soil layers (Fry et al. 2018) (Fig. 4).

The presumably higher proportion of fibrous roots in late successional tree species compared to the higher proportion of taproots in early successional species might have been another driver of high absorptive fine root plasticity in the topsoil of late successional species versus high plasticity in mean rooting depth of early successional species. Root structures with a high proportion of fine roots are considered highly plastic in terms of a shift of location in the soil column especially in the first 10 cm soil depth, while species producing a taproot have less ability to change their morphology and biomass under changes in water availability in grasslands (Fry et al. 2018) and in forests (Yang et al. 2021). Low-cost fibrous roots might enable trees to occupy soil space quickly and more cheaply (in nutrient rich soils), as opposed to the more costly taproots (Fry et al. 2018). Therefore, early successional tree species such as *Larix laricina* might have a greater potential for mean rooting depth plasticity due to faster taproot elongation. This explanation is partially supported by a grassland study where grasses were able to sustain high growth rates during drought periods by increasing plasticity in terms of root topology index (herringbone root architecture) (Hanslin et al. 2019). This change in root topology seemed to be connected to an increase in rooting depth by taproot elongation (Hanslin et al. 2019). However, our results are contrary to earlier studies on trees and shrubs (Olmo et al. 2014) and *poplar* clones (Dickmann et al. 1996). Dickmann et al. (1996) determined slightly higher plasticity in faster growing *poplar* clones in shallow soil layers, while both clones showed high plasticity in terms of fine root growth in deep soil layers without irrigation. The fastest growing trees in Olmo et al. (2014) displayed the lowest root plasticity index in response to no water limitation versus severe drought. These inconsistencies might be caused by the variation in growth forms, species, growth stage, experimental duration, biome, and environmental variables studied (e.g., soil nutrients vs. water availability). Additionally, inconsistencies in results between studies might arise from different classifications of roots, as we separated roots according to function into absorptive, transportive and coarse roots (Fig. 1S6, S7) (McCormack et al. 2015). Further research is needed to better understand the plasticity of fine roots in response to changing water availability conditions depending on root habit and proportion of fibrous roots compared to taproots.

The ability of early versus late successional tree species to show fine root plasticity in deeper soil layers compared to topsoil might also be closely connected to the physical and chemical soil properties that these species encounter in a naturally occurring scenario of disturbance and succession. In support of this interpretation, early successional tree species are generally believed to have greater potential for deep exploitation of a more homogeneous soil substrate, while more shallow rooted late successional species are better adapted to environments that have undergone more soil development thus favoring foraging for nutrients and water close to the soil surface (Gale & Grigal 1987). Early successional tropical tree species have been shown to develop deeper and longer root systems compared to old-growth species in response to drying soil, which allowed them to successfully establish under the warm-dry conditions of secondary succession (Paz et al. 2015). Especially on poor sandy soil, late successional species have been found to allocate proportionally more biomass to roots than early successional species, to presumably extract more nitrogen available in the topsoil (Gleeson & Tilman 1990). However, it has also been hypothesized that early successional species would be more effective at foraging for nutrients in a heterogeneous soil environment, but no relationship between tree successional status and root morphological foraging ability for phosphorous was found (Blair & Perfecto 2004). Further research is needed to elucidate the relationship of fine root foraging ability for late versus early successional tree species in their naturally occurring soil substrates.

## Conclusion

Our study indicates that young *Betula papyrifera*, *Quercus rubra*, *Acer saccharum*, *Larix laricina*, *Pinus strobus*, and *Picea glauca* show acclimation potential to high vs low water availability in terms of fine-root growth. In terms of absorptive fine root biomass and mean rooting depth plasticity, trees follow a two-pronged approach: 1) Late successional species maintain high plasticity in terms of absorptive fine root biomass in the upper soil layer (0–5 cm), while 2) early successional species maintain high plasticity in mean rooting depth. These two different rooting strategies seem to benefit both early and late successional trees since five out of the six tree species did not show any changes in aboveground growth in response to our water treatments (Table S12 & S13). However, as climatic conditions continue to change due to global warming, affecting both precipitation and temperature, water availability will be greatly affected in the future (Masson-Delmotte et al. 2021). Such changes will put pressure on already established trees to modify their fine root acclimation strategy to cope with these changing conditions.

### Supplementary Information

Below is the link to the electronic supplementary material.

Supplementary file1 (PDF 478 KB)

## Data Availability

Data available from the Open Science Framework (OSF) digital repository https://doi.org/10.17605/OSF.IO/6AXCZ.
