# Supplementary material for: Young temperate tree species show different fine root acclimation capacity to growing season water availability
Source: Plant Soil. 2023 Nov 17;496(1-2):485–504. doi: 10.1007/s11104-023-06377-w (PMC10948563; doi:10.1007/s11104-023-06377-w)
Supplement: Supplementary file 1 — Supplementary file1 (PDF 478 KB) [file 11104_2023_6377_MOESM1_ESM.pdf]

Supplementary Information:

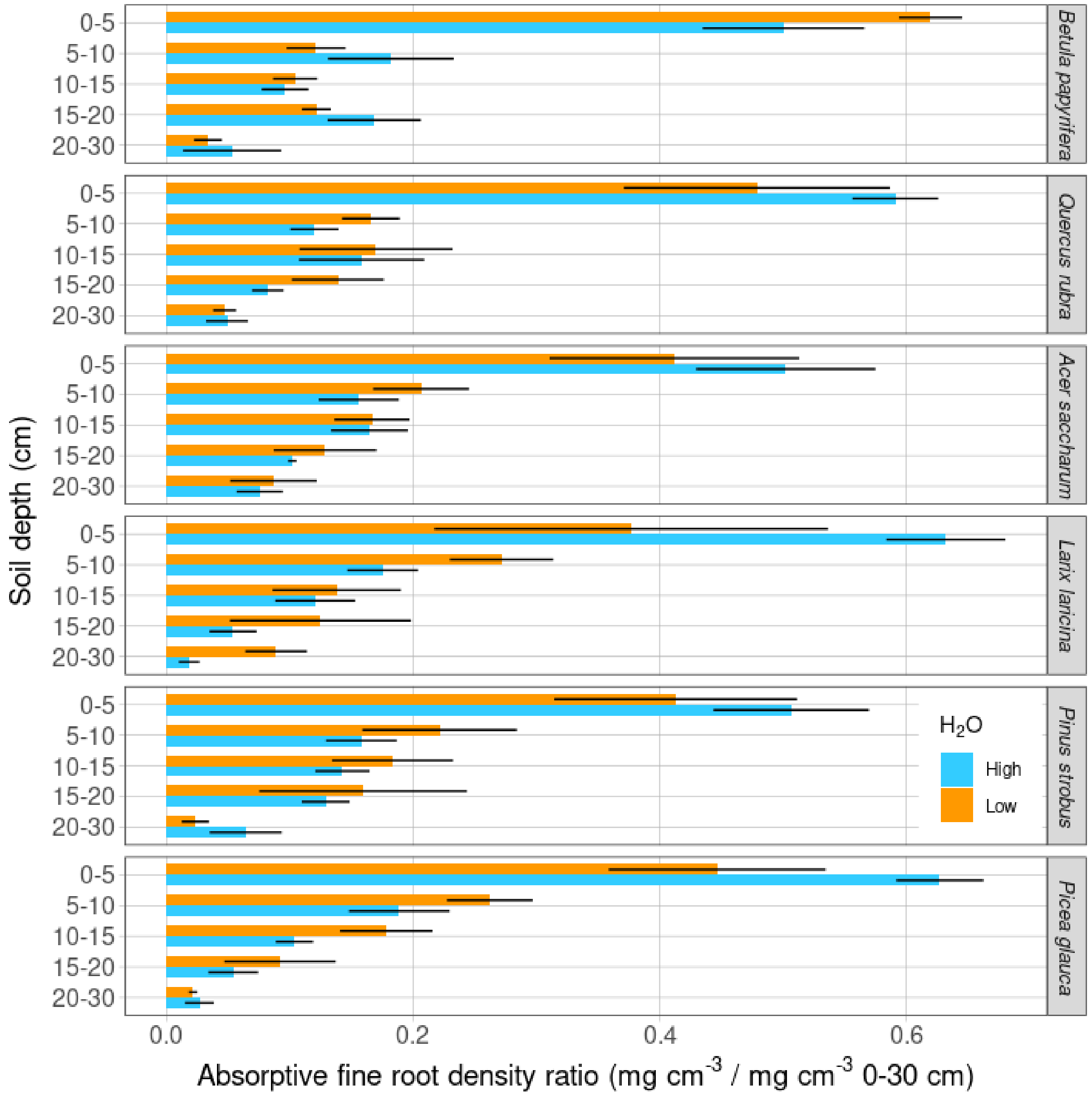

Fig. S1 Variation in mean (± standard error of the mean) absorptive fine root density ratio of each soil depth related to total absorptive fine root density from 0-30 cm soil depth. Soil depths for six tree species in the high (blue) and low (orange) water treatments (H_2O). No statistical

analysis was performed on this ratio. From top to bottom, data for broadleaved and conifer species are presented separately in order of increasing shade tolerance.

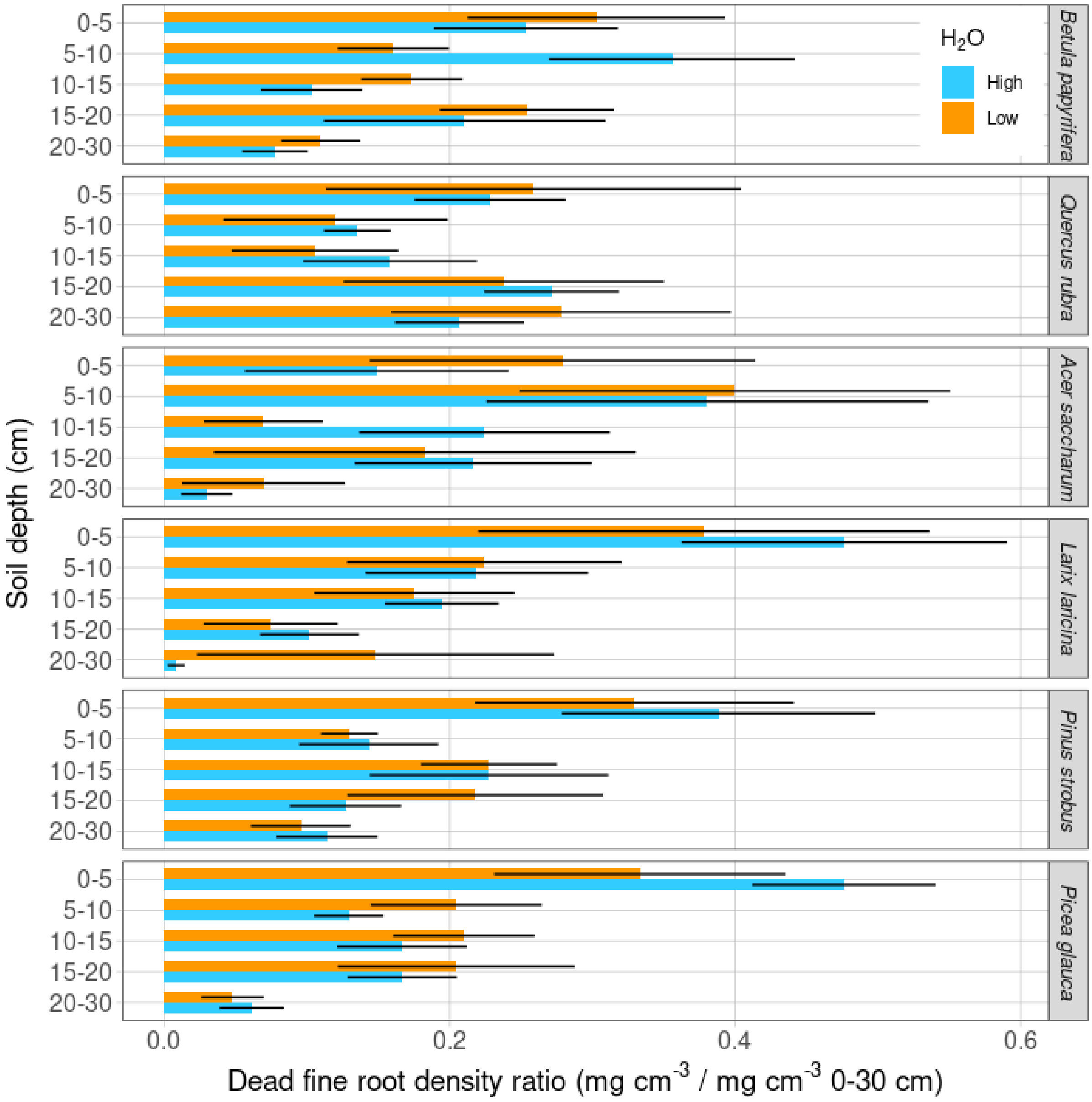

Fig. S2 Variation in mean (± standard error of the mean) dead fine root density ratio of each soil depth related to total absorptive fine root density from 0-30 cm soil depth. Soil depths for six tree species in the high (blue) and low (orange) water treatments (H_2O). No statistical analysis was performed on this ratio. From top to bottom, data for broadleaved and conifer species are presented separately in order of increasing shade tolerance.

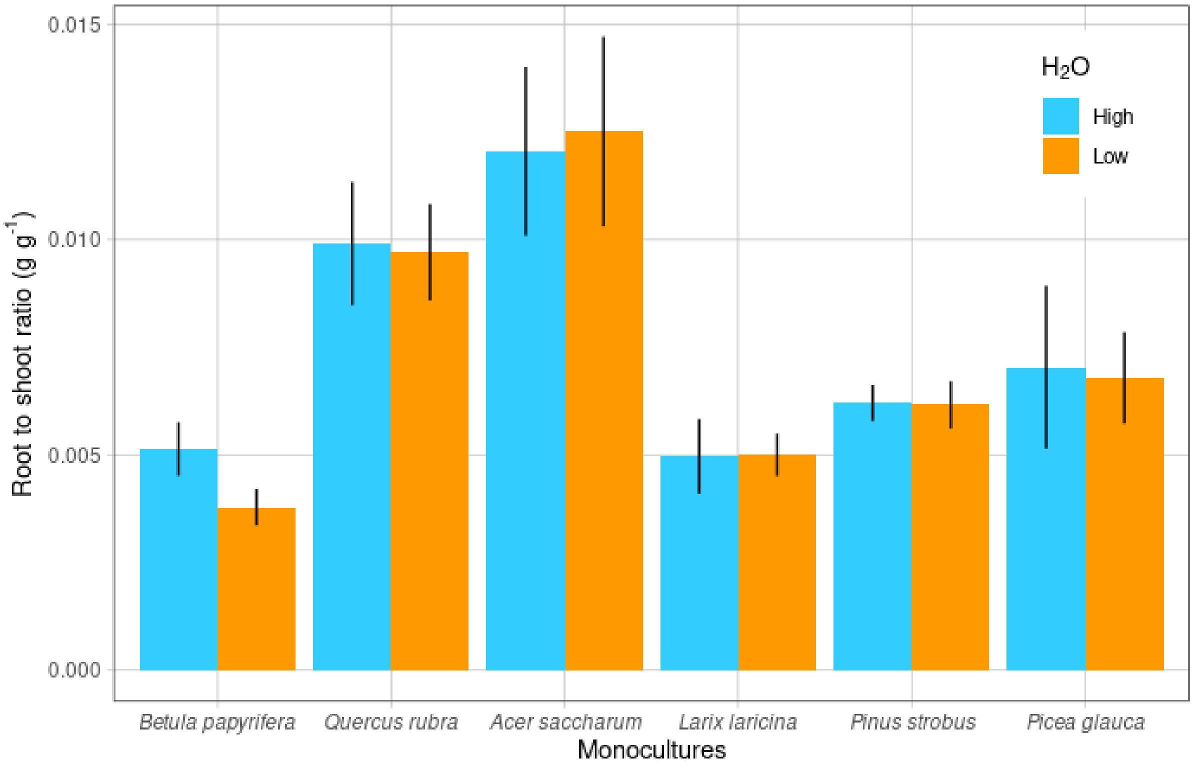

Fig. S3 Variation in mean of root to aboveground biomass ratio (absorptive fine roots, transportive fine roots, and coarse roots), (R/S ratio, ± standard error of the mean) for six tree species in the high (blue) and low (orange) water treatments (H_2O). Significant water treatment effects for a given soil depth are noted by an asterisk and marginal effects by a dot (‘*’, $p< 0.05$ – $p<0.1$ ‘.’). From left to right, data for broadleaved and conifer species are presented separately in order of increasing shade tolerance.

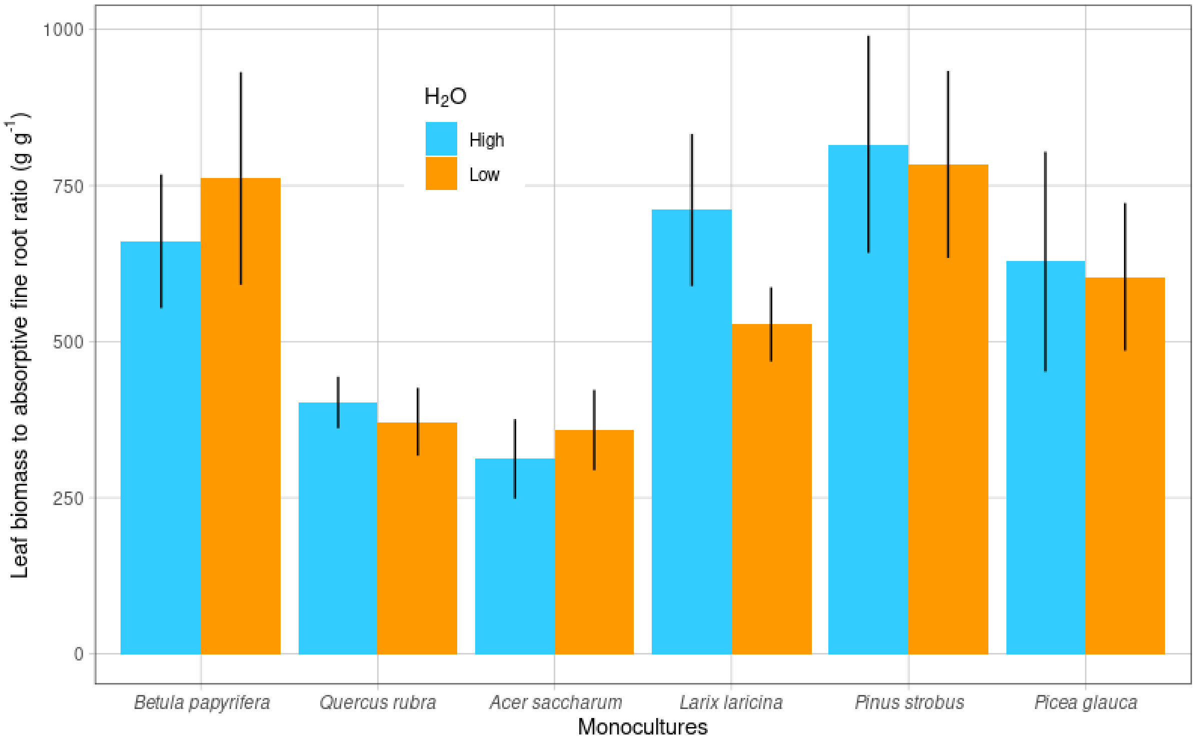

Fig. S4 Variation in mean leaf biomass to absorptive fine root ratio (± standard error of the mean) for six tree species in the high (blue) and low (orange) water treatments (H_2O). Significant water treatment effects for a given soil depth are noted by an asterisk and marginal effects by a dot (‘*’, $p< 0.05$ – $p<0.1$ ‘.’). From left to right, data for broadleaved and conifer species are presented separately in order of increasing shade tolerance.

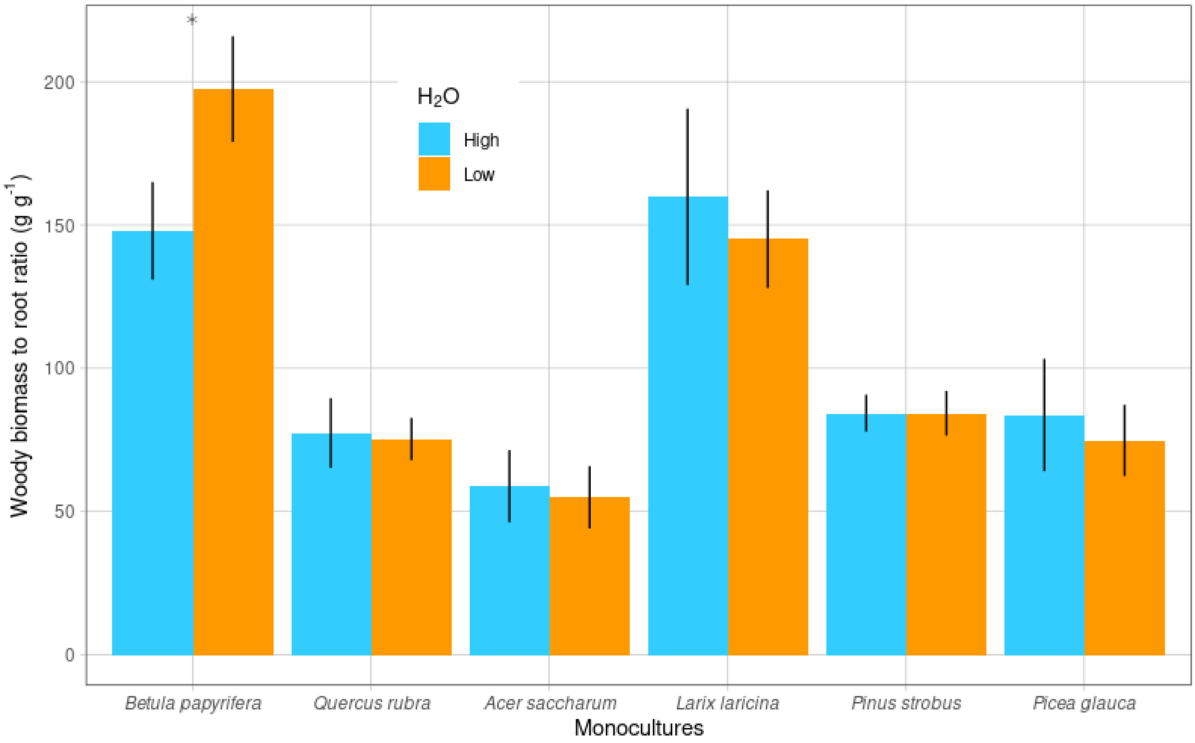

Fig. S5 Variation in mean aboveground woody biomass to root ratio (absorptive fine roots, transportive fine roots, and coarse roots) (± standard error of the mean) for six tree species in the high (blue) and low (orange) water treatments (H_2O). Significant water treatment effects for a given soil depth are noted by an asterisk and marginal effects by a dot (‘*’, $p< 0.05$ – $p<0.1$ ‘.’). From left to right, data for broadleaved and conifer species are presented separately in order of increasing shade tolerance.

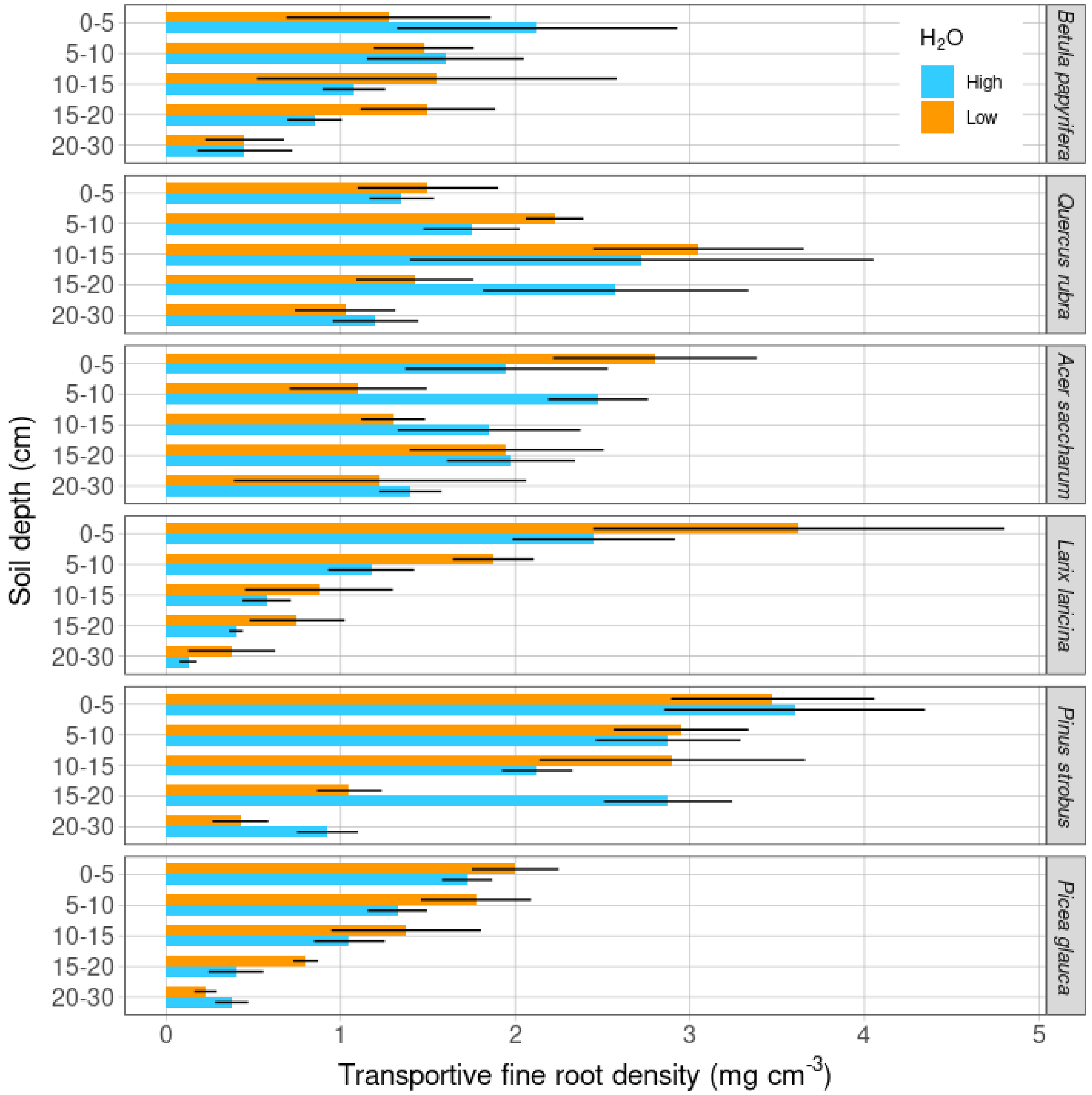

Fig. S6 Variation in mean (± standard error of the mean) transportive fine root density with soil depth for six tree species in the high (blue) and low (orange) water treatments (H_2O). From top to bottom, data for broadleaved and conifer species are presented separately in order of increasing shade tolerance.

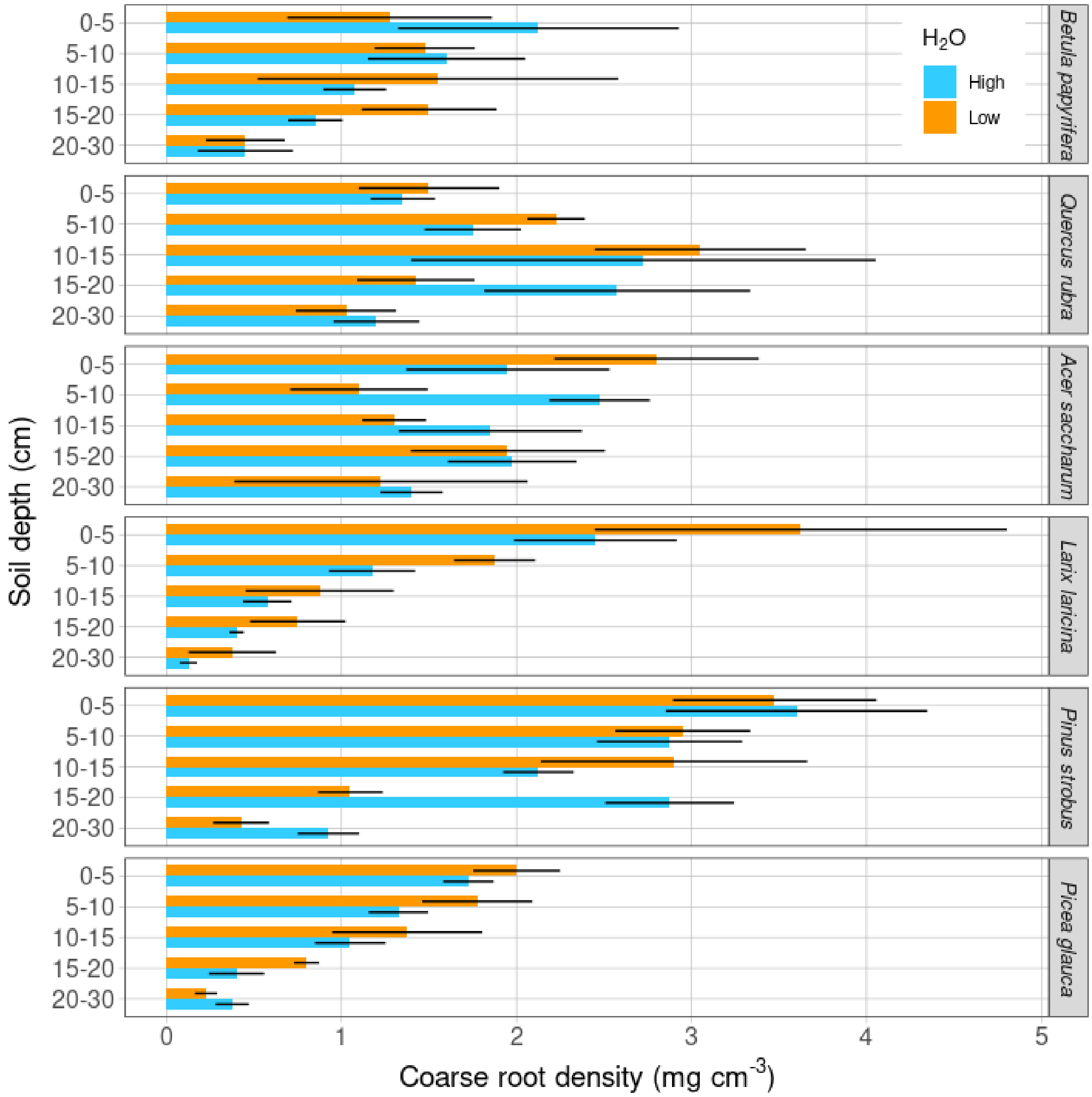

Fig. S7 Variation in mean (± standard error of the mean) coarse root density with soil depth for six tree species in the high (blue) and low (orange) water treatments (H_2O). From top to bottom, data for broadleaved and conifer species are presented separately in order of increasing shade tolerance.

Supplementary tables:

Table S1 Post-hoc Tukey-test results for the interaction between water availability (High vs. Low), species (*Acer saccharum*: As, *Betula papyrifera*: Bp, *Pinus strobus*: Ps, *Picea glauca*: Pg, and *Larix laricina*: Ll), and soil depth (cm) on absorptive fine root density (mg cm^{-3}). Responses between high and low water availability are tested separately for each species and soil depth layer.

Species	Soil depth	Estimate	t-ratio	Pr>[t]

Bp	0 – 5	-0.06	-0.210	0.83
	5 – 10	0.29	0.974	0.33
	10 - 15	0.04	0.133	0.89
	15 – 20	0.28	0.919	0.35
	20 - 30	0.16	0.537	0.59
Qr	0 – 5	0.25	0.823	0.41
	5 – 10	-0.26	-0.849	0.39
	10 - 15	-0.08	-0.261	0.79
	15 – 20	-0.35	-1.140	0.25
	20 - 30	-0.01	-0.034	0.97
As	0 – 5	0.51	1.682	**0.09**
	5 – 10	0.01	0.060	0.95
	10 - 15	0.22	0.747	0.45
	15 – 20	0.15	0.491	0.62
	20 - 30	0.11	0.360	0.71
Ll	0 – 5	0.76	2.494	**<0.05**
	5 – 10	-0.13	-0.450	0.65
	10 - 15	0.21	0.703	0.48
	15 – 20	-0.08	-0.277	0.78
	20 - 30	-0.43	-1.408	0.16
Ps	0 – 5	0.31	1.014	0.31
	5 – 10	-0.12	-0.395	0.69
	10 - 15	-0.05	-0.164	0.87
	15 – 20	0.13	0.452	0.65
	20 - 30	0.41	1.348	0.18
Pg	0 – 5	0.58	1.905	**0.05**
	5 – 10	-0.10	-0.336	0.73
	10 - 15	-0.18	-0.608	0.54
	15 – 20	-0.09	-0.315	0.75
	20 - 30	0.17	0.572	0.56

Table S2 Post-hoc Tukey-test results for the interaction between water availability (High vs. Low), species (*Acer saccharum*: As, *Betula papyrifera*: Bp, *Pinus strobus*: Ps, *Picea glauca*: Pg, and *Larix*

laricina: Ll*)*, and soil depth (cm) on dead fine root density (mg cm^{-3}). Responses between high and low water availability are tested separately for each species and soil depth layer.

**Species**	**Depth**	**Ratio**	**z-ratio**	**Pr>[t]**
Bp	0 – 5	1.76	1.691	**0.09**
	5 – 10	5.56	4.561	**<0.001**
	10 – 15	1.26	0.581	0.56
	15 – 20	1.75	1.589	0.11
	20 – 30	1.55	0.959	0.33
Qr	0 – 5	1.09	0.212	0.83
	5 – 10	0.98	-0.034	0.97
	10 – 15	2.06	1.456	0.14
	15 – 20	0.98	-0.050	0.95
	20 – 30	1.32	0.709	0.47
As	0 – 5	0.46	-1.721	**0.08**
	5 – 10	0.88	-0.330	0.74
	10 – 15	3.62	2.087	**<0.05**
	15 – 20	0.86	-0.324	0.74
	20 – 30	0.34	-1.270	0.20
Ll	0 – 5	1.78	2.069	**<0.05**
	5 – 10	1.88	1.973	**<0.05**
	10 – 15	1.91	1.967	**<0.05**
	15 – 20	2.15	1.875	**0.06**
	20 – 30	0.10	-3.426	**<0.001**
Ps	0 – 5	1.55	1.648	**0.09**
	5 – 10	1.94	2.250	**<0.05**
	10 – 15	1.69	1.881	**0.06**
	15 – 20	1.03	0.114	0.90
	20 – 30	1.96	2.127	**<0.05**
Pg	0 – 5	1.58	1.653	**0.09**
	5 – 10	0.79	-0.706	0.48
	10 – 15	0.69	-1.152	0.24
	15 – 20	0.12	0.350	0.72
	20 – 30	1.60	1.014	0.31

Table S3 Post-hoc test results of the interaction between water availability (High vs. Low) and species (*Acer saccharum*: As, *Betula papyrifera*: Bp, *Pinus strobus*: Ps, *Picea glauca*: Pg, and *Larix laricina*: Ll) on absorptive fine roots weighted mean rooting depth (cm). Contrasts between high and low water availability are tested separately for each species.

Species	Estimate	t-ratio	Pr>[t]
Bp	0.02	0.779	0.44
Qr	-0.02	-0.677	0.50
As	-0.01	-0.481	0.63
Ll	-0.06	-2.006	**0.06**
Ps	-0.002	-0.079	0.93
Pg	-0.04	-1.299	0.21

Table S4 Linear regression (Y = β0 + β1 X + ε) results from bootstrapped (6000 iterations) plasticity indices (PIv, CV, PI:CV, and CVm) of absorptive fine root biomass (g m^{-2} in 0-5 cm soil depth) and aboveground woody biomass (woody biomass, g / tree).

Variable	Estimate	Conf. Int (95 %)	P
PIv (Intercept)	0.5787	0.5744 – 0.5831	**<0.001**
WBIO	-0.0009	-0.0009 – -0.0009	**<0.001**
CV (Intercept)	0.7156	0.7116 – 0.7196	**<0.001**
WBIO	-0.0009	-0.0009 – -0.0009	**<0.001**
PI : CV (Intercept)	0.9086	0.8998 – 0.9173	**<0.001**
WBIO	-0.0007	-0.0007 – -0.0006	**<0.001**
CVm (Intercept)	0.5664	0.5617 – 0.5711	**<0.001**
WBIO	-0.0010	-0.0010 – -0.0010	**<0.001**

Table S5 Linear regression (Y = β0 + β1 X + ε) results from bootstrapped (6000 iterations) plasticity indices (PIv, CV, PI:CV, and CVm) of weighted mean rooting depth (mg cm^{-3}, 0-30 cm soil depth) and aboveground woody biomass (woody biomass, g / tree).

Variable	Estimates	Conf. Int (95 %)	P
PIv (Intercept)	0.0075	0.0046 – 0.0104	**<0.001**
WBIO	0.0006	0.0006 – 0.0006	**<0.001**

CV (Intercept)	0.0642	0.0619 – 0.0665	**<0.001**
WBIO	0.0005	0.0005 – 0.0005	**<0.001**
PI : CV (Intercept)	0.6137	0.6044 – 0.6230	**<0.001**
WBIO	0.0005	0.0004 – 0.0005	**<0.001**
CVm (Intercept)	-0.0173	-0.0199 – -0.0147	**<0.001**
WBIO	0.0005	0.0005 – 0.0005	**<0.001**

Table S6 Results of Type Ⅲ analyses of variance testing for the tree species (*Betula papyrifera*, *Quercus rubra*, *Acer saccharum*, *Larix laricina*, *Pinus strobus*, and *Picea glauca*), and high and low water (H_2O) treatment on root to shoot ratio (R/S ratio, g g^{-1}).

**Source of variation**	**DF**	***F***	**P**
	**Effect on root to shoot ratio (g g^{-1})**		
Species	5	15.14	**<0.001**
H_2O	1	0.04	0.83
Species x H_2O	5	0.15	0.97

Table S7 Post-hoc test results of the interaction between water availability (High vs. Low) and species (*Acer saccharum*: As, *Betula papyrifera*: Bp, *Pinus strobus*: Ps, *Picea glauca*: Pg, and *Larix laricina*: Ll) on root to shoot ratio (R/S ratio, g g^{-1}). Contrasts between high and low water availability are tested separately for each species.

**Species**	**Estimate**	**t-ratio**	**Pr>[t]**
Bp	0.001	0.765	0.45
Qr	0.0002	0.114	0.91
As	-0.0004	-0.260	0.79
Ll	-0.00003	-0.023	0.98
Ps	0.00004	0.025	0.98
Pg	0.0002	0.141	0.88

Table S8 Results of Type Ⅲ analyses of variance testing for the tree species (*Betula papyrifera*, *Quercus rubra*, *Acer saccharum*, *Larix laricina*, *Pinus strobus*, and *Picea glauca*), and high and low water (H_2O) treatment on leaf biomass to absorptive fine root ratio (g g^{-1}).

**Source of variation**	**DF**	***F***	**P**
	**Effect on leaf biomass to absorptive fine root ratio (g g^{-1})**		
Species	5	6.43	**<0.001**
H_2O	1	0.04	0.84
Species x H_2O	5	0.44	0.81

Table S9 Post-hoc test results of the interaction between water availability (High vs. Low) and species (*Acer saccharum*: As, *Betula papyrifera*: Bp, *Pinus strobus*: Ps, *Picea glauca*: Pg, and *Larix laricina*: Ll) on leaf biomass to absorptive fine root ratio (g g^{-1}). Contrasts between high and low water availability are tested separately for each species.

**Species**	**Estimate**	**t-ratio**	**Pr>[t]**
Bp	-100.8	-0.625	0.54
Qr	31.2	0.194	0.84
As	-46.4	-0.288	0.77
Ll	183.1	1.136	0.27
Ps	32.1	0.199	0.84
Pg	24.5	0.152	0.88

Table S10 Results of Type Ⅲ analyses of variance testing for the tree species (*Betula papyrifera*, *Quercus rubra*, *Acer saccharum*, *Larix laricina*, *Pinus strobus*, and *Picea glauca*), and high and low water (H_2O) treatment on aboveground woody biomass to root ratio (g g^{-1}) (absorptive fine roots, transportive fine roots, and coarse roots).

**Source of variation**	**DF**	***F***	**P**
	**Effect on woody biomass to root mass ratio (g g^{-1})**		
Species	5	24.30	**<0.001**

H_2O	1	0.05	0.82
Species x H_2O	5	1.48	0.22

Table S11 Post-hoc test results of the interaction between water availability (High vs. Low) and species (*Acer saccharum*: As, *Betula papyrifera*: Bp, *Pinus strobus*: Ps, *Picea glauca*: Pg, and *Larix laricina*: Ll) on aboveground woody biomass to root ratio (g g^{-1}) (absorptive fine roots, transportive fine roots, and coarse roots). Contrasts between high and low water availability are tested separately for each species.

**Species**	**Estimate**	**t-ratio**	**Pr>[t]**
Bp	-49.55	-2.219	**<0.05**
Qr	2.013	0.090	0.92
As	3.892	0.174	0.86
Ll	14.772	0.661	0.51
Ps	0.029	0.001	0.99
Pg	8.915	0.399	0.69

Table S12 Results of Type Ⅲ analyses of variance testing for the tree species (*Betula papyrifera*, *Quercus rubra*, *Acer saccharum*, *Larix laricina*, *Pinus strobus*, and *Picea glauca*), and high and low water (H_2O) treatment on aboveground woody biomass (g).

**Source of variation**	**DF**	***F***	**P**
	**Effect on woody biomass (g)**		
Species	5	31.66	**<0.001**
H_2O	1	9.14	**<0.05**
Species x H_2O	5	0.36	0.87

Table S13 Post-hoc test results of the interaction between water availability (High vs. Low) and species (*Acer saccharum*: As, *Betula papyrifera*: Bp, *Pinus strobus*: Ps, *Picea glauca*: Pg, and *Larix laricina*: Ll) on aboveground woody biomass (g). Contrasts between high and low water availability are tested separately for each species.

**Species**	**Estimate**	**t-ratio**	**Pr>[t]**
Bp	68.2	1.673	0.11

Qr	42.0	1.031	0.31
As	50.7	1.244	0.23
Ll	105.3	2.584	**<0.05**
Ps	78.1	1.917	**0.07**
Pg	69.6	1.709	**0.10**
